# Human multipotent mesenchymal stromal cells cytokine priming promotes RAB27B-regulated secretion of small extracellular vesicles with immunomodulatory cargo

**DOI:** 10.1186/s13287-020-02050-6

**Published:** 2020-12-14

**Authors:** Anastasia Cheng, Dongsic Choi, Maximilien Lora, Dominique Shum-Tim, Janusz Rak, Inés Colmegna

**Affiliations:** 1 Research Institute of the McGill University Health Centre, McGill University, 1001 Decarie Blvd, Office # EM2-3238, Montreal, QC H4A 3J1 Canada; 2 Division of Cardiac Surgery, Department of Surgery, McGill University, Montreal, QC Canada; 3 Division of Rheumatology, Department of Medicine, McGill University, Montreal, QC Canada

**Keywords:** Multipotent mesenchymal stromal cells, MSC, Extracellular vesicles, Exosomes, sEVs, RAB27B, TSG6, A20

## Abstract

**Background:**

The paracrine effects of multipotent mesenchymal stromal cells (MSCs) are mediated by their secretome composed by soluble factors (i.e., cytokines, growth factors, hormones) and extracellular vesicles (EVs). EVs promote intercellular communication, and the EV cargoes [e.g., proteins, soluble factors, microRNAs (miRNAs), messenger RNA (mRNA), DNA] reflect the molecular and functional characteristics of their parental cells. MSC-derived EVs (MSC-EVs) are currently evaluated as subcellular therapeutics. A key function of the MSC secretome is its ability to promote immune tolerance (i.e., immunopotency), a property that is enhanced by priming approaches (e.g., cytokines, hypoxia, chemicals) and inversely correlates with the age of the MSC donors. We evaluated mechanisms underlying MSC vesiculation and the effects of inflammation and aging on this process.

**Methods:**

We evaluated the effects of interferon gamma (IFN-γ) and tumor necrosis factor alpha (TNF-α) on human adipose-derived MSC: (a) vesiculation (custom RT^2^ Profiler PCR Array), (b) EV profiles (Nanoparticle Tracking Analysis and Nanoparticle Flow Cytometry), (c) EV cargo (proteomic analysis and Western blot analysis), and (d) immunopotency (standard MSC:CD4 T cell proliferation inhibition assay). We confirmed the role of RAB27B on MSC vesiculation (RAB27B siRNA) and assessed its differential contribution to vesiculation in adult and pediatric MSCs (qPCR).

**Results:**

Cytokine priming upregulated *RAB27B* in adipose-derived MSCs increasing their secretion of exosome-like small EVs (sEVs; < 200 nm) containing two key mediators of immunopotency: A20 and TSG-6. These EVs inhibited T cell proliferation in a dose-dependent manner. *RAB27B* siRNA inhibited MSC vesiculation. Adipose-derived MSCs isolated from pediatric donors exhibited higher RAB27B expression and secreted more sEVs than adult MSCs.

**Conclusions:**

Cytokine priming is a useful strategy to harvest anti-inflammatory MSC-sEVs for clinical applications. Of relevance, donor age should be considered in the selection of MSC-sEVs for clinical applications.

## Background

Mesenchymal stromal cells (MSCs) are a heterogeneous group of multipotent progenitor cells that can be isolated from adult tissues [e.g., bone marrow (BM), adipose (AT)]. MSCs possess functional properties (e.g., high expansion capacity, low immunogenicity, anti-inflammatory/pro-angiogenic, and anti-fibrotic effects) that motivate their use in ongoing clinical trials of immune-mediated, inflammatory, and degenerative diseases [1, 2]. A *sine-qua-non* to enhance the effectiveness of MSC therapy is the understanding of their biology and mechanisms of action, as well as those approaches which potentiate desired MSC functions [3, 4].

MSCs display limited engraftment or persistence capacity at sites of injury [5–7]. The functional improvements post-MSC infusion are in part mediated by paracrine mechanisms (e.g., cytokines, chemokines, and growth factors) which modulate the MSC microenvironment and influence the activity of resident cells [8]. In fact, MSC-conditioned media (MSC CM) recapitulates many effects of MSCs and is an alternative to the use of cell therapy [9–13]. The MSC secretome is highly dynamic due to its ability to sense and respond to environmental cues [14]. For example, pro-inflammatory cytokines (e.g., IFN-γ, TNF-α, IL-1) change the content of immunomodulatory factors secreted by MSCs (e.g., TSG-6, PGE_2_, HGF, TGF-β1, and IL-10) [15–17]. Hypoxic conditions, on the other hand, stimulate MSCs to secrete pro-angiogenic factors (e.g., IGF-1, TGF-β1, VEGF, angiogenin, EGF, and bFGF) [18].

Extracellular vesicle (EV) production is an evolutionarily conserved process fundamental for the removal of intracellular “surplus” molecules and for intercellular communication [19, 20]. Secreted EVs carry complex assemblies of bioactive factors (i.e., cargo) such as lipids, proteins (transcription factors, growth factors, and enzymes), nucleic acids (RNAs: mRNAs, microRNAs-miRNAs, and non-coding RNAs-lncRNAs; and DNA: ssDNA and dsDNA), and in some cases components of organelles (e.g., mitochondrial DNA) [21, 22]. EVs are heterogeneous and so are the emerging pathways of their biogenesis with the emerging roles of endosomal sorting complex required for export (ESCRT), GTPases (RAB7, RAB11, RAB27A, RAB27B), sphingomyelinases (SPMD3), tetraspanins (CD63, CD81), and other mechanisms [21]. Upon uptake, the EV cargo modulates the activity of recipient cells [23–25]. EVs secreted by MSCs (MSC-EVs) are a promising therapeutic component of the MSC secretome [26]. Most preclinical studies involving MSC-EV therapy are based on vesicles produced by resting MSCs [27, 28]. Little is known about how the local microenvironment influences MSC vesiculation.

The functional properties of MSCs are influenced by the donor’s age and age-associated diseases (e.g., atherosclerosis, type 2 diabetes) [29, 30]. We showed that these characteristics alter the secretome and reduce the immunosuppressive ability of MSCs [29, 30]. In addition, multiple plasma EV populations associated with immune cells decline with aging, while age-associated chronic diseases may additionally alter EV production [31–33], but rigorous studies involving MSC-EVs are lacking. In this study, we addressed the effects of cytokine priming and donor age on human AT-MSC vesiculation. We showed that cytokine priming upregulates RAB27B, increasing the secretion of small EVs (sEVs; < 200 nm) containing A20 and TSG-6, key mediators of MSC immunopotency. Accordingly, these EVs inhibit T cell proliferation in a dose-dependent manner. We also found that MSCs from young donors display higher *RAB27B* expression and secrete more sEVs relative to MSCs from older donors. Our study suggests that, cytokine priming and aging impact the quantity and cargo of MSC-EVs, thus these factors need to be considered in preparing EVs for clinical trials.

## Methods

### Study subjects

This study was approved by the McGill University Health Center Ethics Review Board (Protocol 10-107). All participants provided written informed consent. Subcutaneous adipose tissue was obtained from 9 pediatric (Ped; 16.1 ± 2.6 years) and 14 adult donors (Ad; 62.6 ± 12.7 years). Table S1 summarizes the demographic characteristics of the study participants.

### Human AT-MSC isolation and expansion

Human AT-MSCs were isolated and expanded as previously described and characterized according to the minimal definition criteria suggested by the International Society for Cellular Therapy (ISCT) [29, 34] (Fig. S1). Briefly, human subcutaneous adipose tissue samples were washed with phosphate-buffered saline (PBS), minced with surgical scissors, and digested with 0.05% collagenase (Millipore Sigma) in Hank’s balanced salt solution (HBSS) (Invitrogen). The enzyme was then neutralized with 5% Gibco® MSC Qualified fetal bovine serum (FBS; Thermo Fisher Scientific). Dissociated cells were collected by centrifugation (4 °C, 800*g* for 5 min) and re-suspended in complete medium (1.0 g/L glucose, with L-glutamine and sodium pyruvate Dulbecco’s modified Eagle’s medium [DMEM; WISENT Inc.]) supplemented with 10% FBS and 1% penicillin/streptomycin (10,000 units/mL penicillin, 10,000 mg/mL streptomycin, WISENT Inc.). MSCs were seeded at 1 g of tissue/flask and cultured under standard conditions (5% carbon dioxide, 37 °C) in 75-cm^2^ tissue culture flasks. Two days after the initial isolation, non-adherent-cells were washed off and complete media was replaced. When MSCs reached 80% confluency, they were detached with 0.25% Trypsin-EDTA (37 °C for 5 min) and re-seeded at a density of 5000 cells/cm^2^. Passage 1 MSCs were stored in liquid nitrogen. Passage 4 MSCs were used for all experiments.

### MSC CM preparation

MSCs (2.5 × 10^5^ cells/ml) were preconditioned/primed for 72 h with IFN-γ (10 ng/mL, R&D Systems) and TNF-α (15 ng/mL, R&D Systems) as suggested by the ISCT [35]. MSC CM was prepared in EV collection media [phenol red-free low-glucose DMEM containing 1% insulin-transferrin-selenium (ITS; Thermo Fisher Scientific)]. This media did not contain serum (e.g., fetal bovine serum—FBS) to avoid contamination with serum microparticles [36]. Resting MSCs (not primed with cytokines) served as controls. After removing cells and debris by centrifugation, MSC CM were stored at − 80 °C.

### MSC immunopotency assay

Peripheral blood mononuclear cells (PBMCs) were isolated from one healthy donor (26 years old female) with Lymphocyte Separation Medium (Mediatech, Inc.) through density gradient centrifugation. PBMCs were stained with carboxyfluorescein succinimidyl ester (CFSE; Millipore Sigma) and stimulated with Concanavalin A (ConA; 10 μg/ml) in Rosewell Park Memorial Institute medium (RPMI-1640) (WISENT Inc.) supplemented with 10% FBS and 1% Penicillin Streptomycin. CFSE-stained and ConA-activated PBMCs (2 × 10^6^, 100 μl) were added to either (a) resting/primed MSC CM (100 μl) or (b) MSC-EVs (2 or 4 μg in 100 μl). Activated PBMCs cultured in EV collection media served as the positive control (maximal proliferation—Max), while non-activated PBMCs served as a negative control (minimal proliferation—Min). After 4 days, the PBMCs were collected and stained with 7-aminoactinomycin D (7-AAD, Cat# 559925) and CD4-APC (Cat# 555349; BD Biosciences). The Expansion Index (EI) of 7AAD^−^/CD4^+^ cells (viable CD4^+^ T cells) was determined with FlowJo software. The proliferation of CD4^+^ T cells was calculated using the following formula:

$$ \mathrm{CD}{4}^{+}\ \mathrm{T}\ \mathrm{cell}\ \mathrm{proliferation}\ \left(\%\right)=\frac{\left(x-\operatorname{Min}\right)}{\operatorname{Max}-\operatorname{Min}}\times 100\% $$

where *x* = EI of stimulated CD4^+^ T cells in the presence of MSC or derived products; Min = EI of non-stimulated CD4^+^ T cells; and Max = EI of stimulated CD4^+^ T cells in the absence of MSCs.

### Collection of EVs from MSC CM

EVs were obtained from MSC CM as previously described with some modifications [37] and were characterized according to recommendations of the International Society for Extracelular Vesicles (ISEV) [20]. MSCs were grown in T75-cm^2^ flasks with complete medium until they reached 80% confluency. MSCs were then washed with PBS and cultured an additional 3 days in EV collection medium with or without cytokines for priming. Cells and debris were eliminated from the CM by centrifugation at 800*g* for 5 min and 2000*g* for 10 min. The CM was then filtered using 0.22-mm pore filters (Millipore Sigma) to eliminate large EVs. EVs were then harvested from the filtered CM by ultracentrifugation at 110,000*g* (Type 70Ti fixed angle, Beckman Coulter) for 2 h at 4 °C. The pellet was washed in 18 ml of PBS prior to a second ultracentrifugation step (4 °C, 110,000*g* for 2 h) and re-suspended in 100 μl of PBS. Samples were stored in 10 μl aliquots at − 80 °C to avoid freeze thawing.

### Nanoparticle tracking analysis

The size and concentration of nanoparticles in MSC CM and EV preparations was measured using NanoSight NS500 (NanoSight) [38]. PBS was used to dilute MSC CM (1:12) and EV preparations (1:100–500) to achieve 40–100 particles per frame during quantification. Five 30-s videos were obtained at room temperature for each sample.

### Quantitative PCR–MSC vesiculation

The expression of genes linked to cellular vesiculation was analyzed in resting and primed MSC (*n* = 6 per group) by real-time PCR using a custom RT^2^ Profiler PCR Array (SABiosciences, Qiagen) as previously described [39]. P4 MSCs were expanded in 75-cm^2^ flasks to 80% confluence in complete medium, washed with PBS, and cultured for an additional 72 h in EV collection medium with or without cytokine priming. TRIzol reagent (Thermo Fisher Scientific) was used as a lysis buffer to extract total RNA from MSC. Purified RNA was obtained using Direct-zol™ RNA MiniPrep (Zymo Research) and quantified using BioDrop μlite spectrophotometer (Harvard Bioscience, Holliston, MA). Reverse transcription (RT) was performed using 1 μg of purified RNA and QuantiTect reverse transcription kit (Qiagen) according to manufacturer’s instructions. Following RT, the complementary deoxyribonucleic acid (cDNA) was combined with RT^2^ SYBR Green qPCR Master Mix (SABiosciences, Qiagen), and 25 μl of this mix was loaded per well of the custom RT^2^ Profiler PCR Array. Quantitative real-time PCR was performed using LightCycler® 96 System software (Roche Molecular Systems) as follows: a 10-min activation step (95 °C), followed by a two-step amplification step for 45 cycles (15 s at 95 °C and 1 min at 60 °C), and a final thermal dissociation step (95 °C 10 s, 65 °C 60 s, 97 °C 1 s). The relative expression of EV-related genes was normalized to the expression of three housekeeping genes (ACTB, GAPDH, YBX1) and calculated using the ΔΔCt method by Schmittgen and Livak [40]. Statistical differences between resting and primed MSCs were determined using 2-way ANOVA with Bonferroni’s correction for multiple comparisons.

### Iodixanol/Optiprep gradient

EV density was assessed using an Iodixanol/Optiprep gradient protocol [41]. Briefly, to assess the density of isolated EVs, EV preparations were loaded in a gradient consisting of 2.5 ml 5%, 3 ml 20%, and 4.5 ml 30% iodixanol (OptiPrep™ Density Gradient Medium, Millipore Sigma) prepared in diluent (0.25 M sucrose, 150 mM NaCl, 20 mM HEPES, pH 7.4). The gradient was separated by ultracentrifugation in an SW-41 Ti rotor (Beckman Coulter) (4 °C, 200,000*g* for 2 h). One milliliter fractions were collected from which 8 μl was retained for NTA. Using a refractometer, the index of refraction for each fraction was measured in the control gradient. Densities were calculated according to the manufacturer’s instructions.

### Transmission electron microscopy of MSC-EVs

TEM images of MSC-EVs were obtained as previously described [42]. Briefly, EVs purified by ultracentrifugation were suspended in EV buffer (137 mM NaCl, 20 mM HEPES, pH 7.5) and fixed onto carbon-coated grids with glutaraldehyde. After fixation, the grids were stained with uranyl acetate and imaged using TEM.

### Western blot

MSC lysates (10^7^ MSC/mL) were prepared in RIPA buffer (50 mM Tris-HCl, 150 mM NaCl, 1% NP-40, 0.5% sodium deoxycholate, and 0.1% SDS, pH 7.4; Boston Bioproducts) and 1× Protease Arrest™ (G-Biosciences). The protein concentrations of cell lysate, CM and EV preparations were quantified using Micro BCA™ Protein Assay Kit (Thermo Fisher Scientific). Fifty micrograms of MSC lysate, MSC CM, MSC-EV, and Page Ruler™ Prestained Protein Ladder (Thermo Fisher Scientific) were loaded into a standard 12% SDS-PAGE and transferred to a PVDF membrane. The membrane was probed with antibodies against CD81 (Thermo Fisher Scientific), ALIX (Santa Cruz Biotechnology), calnexin (Abcam), TSG-6 (Santa Cruz Biotechnology), A20 (Cell Signaling Technology), or GAPDH (Santa Cruz Biotechnology); followed by specific IgG horseradish peroxidase-conjugated antibodies (Jackson ImmunoResearch Labs). Immunoreactive proteins were visualized with Amersham™ ECL Prime Western Blotting Detection Reagent (GE Healthcare Life Sciences) and imaged using Omega Lum™ C Imaging System (Aplegen®).

### Proteomic analysis of MSC-sEVs

Proteomic analysis of MSC-sEVs was performed by the Proteomics Platform of The Research Institute of the McGill University Health Centre (RI-MUHC). For each resting or primed MSC-sEV preparation (*n* = 3 for each group, 5 μg per sample), a single stacking gel band was reduced with DTT, alkylated with iodoacetic acid, and then digested with trypsin. Peptides were re-solubilized in 0.1% aqueous formic acid/2% acetonitrile, loaded onto a Thermo Acclaim Pepmap (Thermo, 75 μM ID × 2 cm C18 3 μm beads) precolumn, and then onto an Acclaim Pepmap Easyspray (Thermo, 75 μM × 15 cm with 2 μm C18 beads) analytical column separation using a Dionex Ultimate 3000 uHPLC at 220 nL/min with a gradient of 2–35% organic (0.1% formic acid in acetonitrile) over 3 h. Peptides were analyzed with a Thermo Orbitrap Fusion mass spectrometer operating at a 120,000 resolution (FWHM in MS1, 15,000 FWHM for MS/MS) with HCD sequencing of all peptides with a charge of 2+ or greater. The raw data was converted into *.mgf format (Mascot generic format), searched using Mascot 2.3 against human sequences (Swissprot 2017). The database search results were loaded onto Scaffold Q+ Scaffold_4.7.2 (Proteome Sciences) for spectral counting statistical treatment and data visualization. Classification of MSC small exosome-like EV cargo by Gene Ontology (GO) cellular component and biological process was performed using the Database for Annotation, Visualization and Integrated Discovery (DAVID) of the National Institute of Allergy and Infectious Disease (http://david.abcc.ncifcrf.gov). Proteomic datasets are freely available upon request to the corresponding author.

### MSC RAB27B silencing

*RAB27B* siRNA (Accell Smart pool, E-004228-00-0005, Dharmacon) was used for gene knockdown in MSC according to the manufacturer’s instructions. Non-targeting siRNA (Accell Non-targeting Pool, D-001910-10, Dharmacon) were used for siRNA control. Briefly, 5 × 10^3^ MSC were seeded in each well of a 96-well plate with complete medium overnight prior to incubation with 100 μL of each siRNA mixture (1 μM) diluted in EV collection medium. After 24 h, the supernatant was discarded, and the cells were washed with PBS before incubation with EV collection medium with or without IFN-γ (10 ng/mL) and TNF-α (15 ng/mL) for 72 h.

### Statistical analysis

All statistical analyses were performed with GraphPad Prism software (GraphPad Prism 8) and presented as bar graphs. Non-parametric tests (Mann-Whitney or Wilcoxon signed-rank test) were used for between groups’ comparisons. All hypotheses tests were 2-sided. A *p* value of < 0.05 was considered statistically significant.

## Results

### Cytokine priming enhances the immunopotency and increases exosome-like EV release by MSCs

**Fig. 1:**
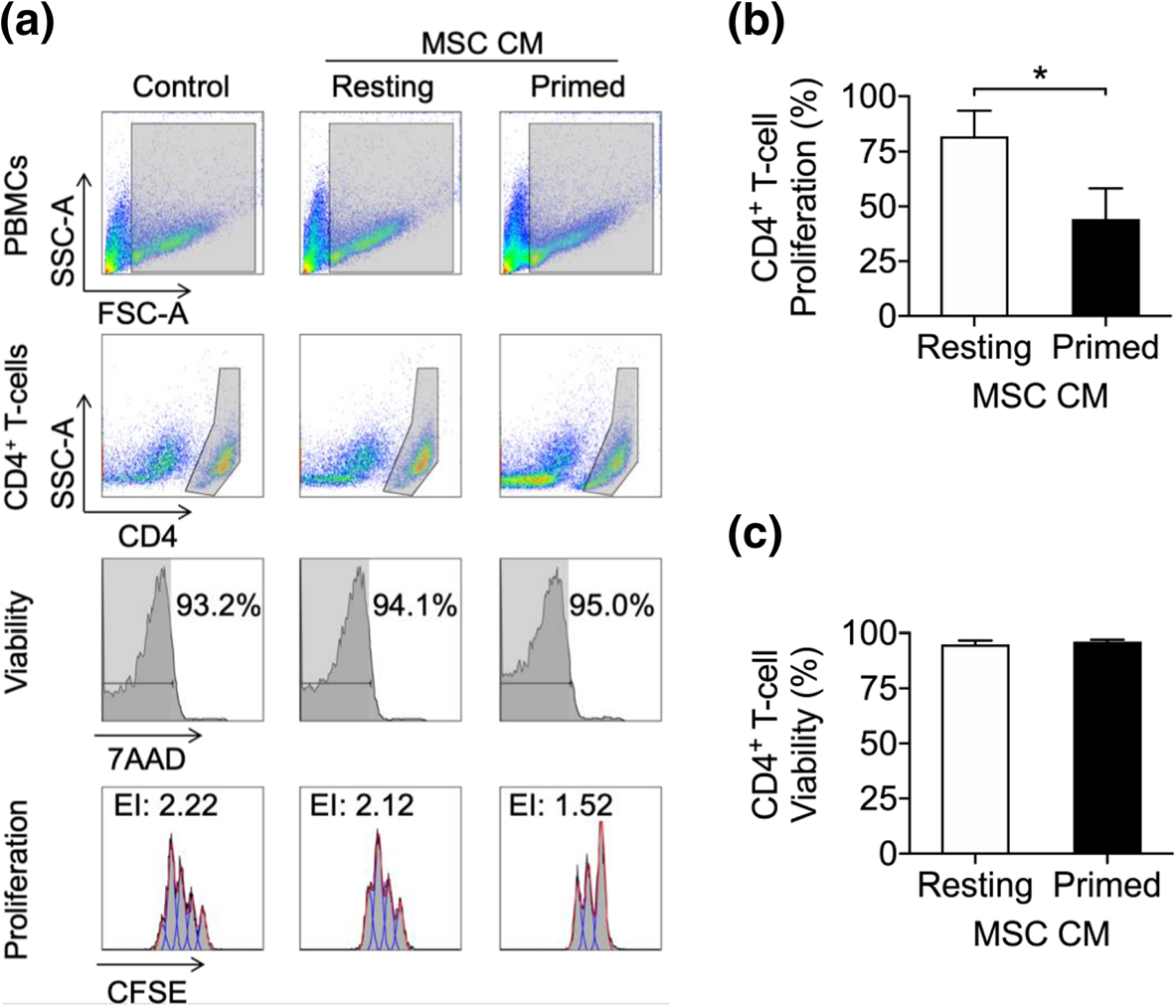
Cytokine priming enhances the ability of MSC-conditioned media (MSC CM) to suppress T cell proliferation. **a** ConA-stimulated T cell proliferation assay with resting and primed MSC CM (representative example). Gating strategy and Expansion Index (EI) of viable CD4^+^ T cells cultured with media conditioned by resting or primed MSC. **b** Summary graph of ConA-stimulated CD4^+^ T cell proliferation and **c** viability in the presence of resting or primed MSC CM. Wilcoxon signed-rank test, mean ± SD of 6 independent samples are reported, (*) represents *p* ≤ 0.05

In order to quantitatively evaluate the effect of cytokine priming on the immunopotency of the MSC secretome, we designed an in vitro assay the sensitivity of which allowed us to test the immunosuppressive ability of MSC CM against T cell targets (section “MSC immunopotency assay”). Using this approach, we observed that IFN-γ and TNF-α-priming enhanced the capacity of AT-MSC CM to suppress the proliferation of activated CD4^+^ T cells compared to CM from resting MSCs (Fig. 1a–b). Of relevance, MSC priming did not alter CD4^+^ T cell viability (Fig. 1c).

**Fig. 2:**
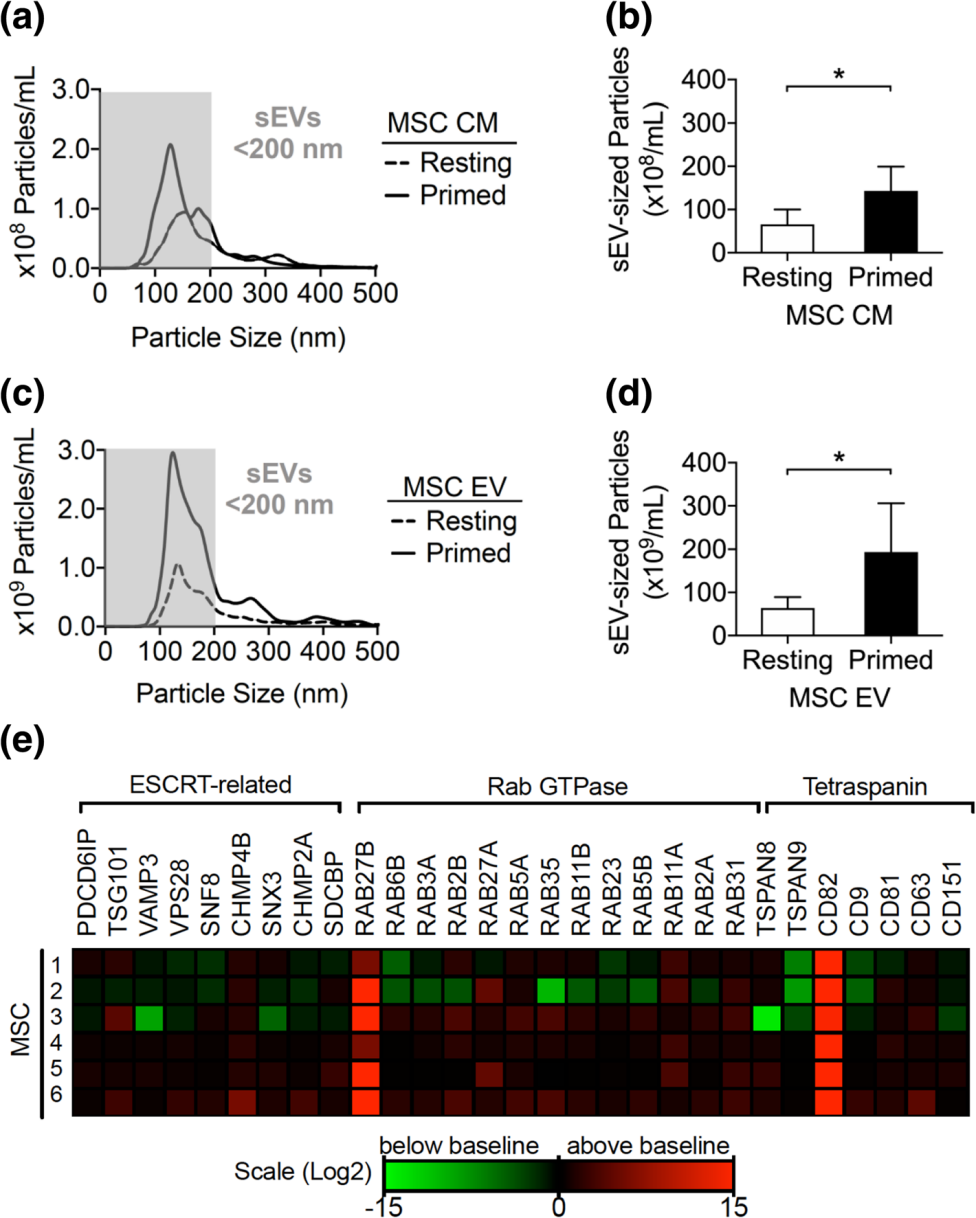
Cytokine priming increases MSC-sEV release. **a** Particle concentration and size distribution of resting and primed MSC CM. Curves represent the average of 6 independent samples. The gray box indicates small EV (sEV)-sized particles (< 200 nm) as defined by ISEV. **b** Quantity of sEV-sized particles in resting and primed MSC CM. **c** Particle concentration and size distribution of EVs collected by filtration and ultracentrifugation of resting and primed MSC CM (curves represent average of 6 independent samples, gray box highlights sEV-sized particles). **d** Quantity of sEV-sized particles in EVs collected from resting and primed MSC CM. **e** Heatmap of vesiculation genes differentially expressed upon MSC priming. Samples 1, 2, and 3 are from pediatric MSC donors and the rest from adult MSC donors. Data represents fold change relative to resting MSC. Increased expression (red) and reduced expression (green) are shown on a log2-based scale. Wilcoxon signed-rank test, mean ± SD of 6 independent samples are reported, (*) represents *p* ≤ 0.05

Because the biological activity of MSC secretome is often attributed to the EV content, we assessed the effect of MSC cytokine priming on EV release by comparing the size distribution and concentration of nanoparticles in the MSC CM using nanoparticle tracking analysis (NTA). Following priming, the number of small particles significantly increased in MSC CM (Fig. 2a,b). This effect required a combined presence of both IFN-γ and TNF-α and was not observed when each of these cytokines was used alone (Fig. S2). Since NTA cannot distinguish protein aggregates, or solid particles from EVs, this analysis was repeated following purification of MSC-EVs by differential centrifugation [37]. Once again, higher numbers of events were observed in the pelleted CM fraction expected to contain small EVs (sEV) and collected by ultracentrifugation (100,000×*g* = 100 K) of primed MSCs versus their resting controls (Fig. 2c,d). No significant changes were observed in MSC viability after cytokine priming, suggesting that the sEVs were not apoptotic/necrotic fragments or cell debris (Fig. S3).

In order to explore possible reasons for the increase in primed MSC vesiculation, we performed a comparative targeted gene expression analysis. Resting and primed MSCs were screened for mRNA levels of EV biogenesis-related genes including ESCRT machinery, Rab GTPases, and tetraspanins (Fig. 2e) [39]. Compared to resting MSCs, *RAB27B* and *CD82* were the only two genes highly upregulated in primed MSCs (19.6-fold and 21.5-fold respectively). The fact that primed MSCs upregulated *RAB27B*, and increased the secretion of sEVs with a density of 1.13 g/mL (section “Primed MSC-EVs carry unique immunomodulatory proteins and inhibit T cell proliferation”), suggest that priming may stimulate biogenesis of particles that resemble and include exosomes [43]. Thus, cytokine priming stimulates the immunomodulatory properties of the MSC secretome along with its sEV content and activity of the vesiculation pathway.

**Fig. 3:**
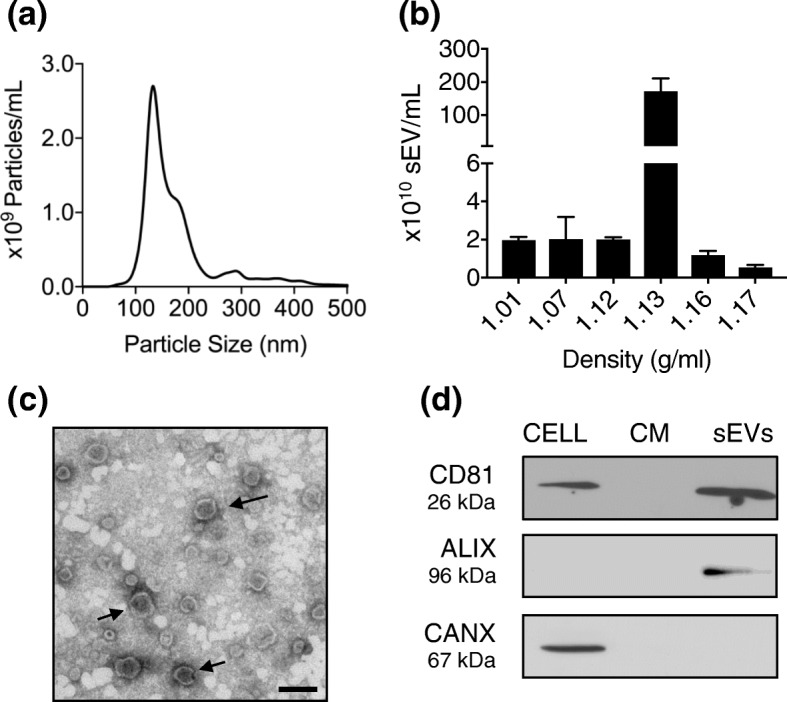
Characterization of cytokine-primed MSC-sEVs. **a** Nanoparticle tracking analysis (NTA) of primed MSC-sEVs. **b** NTA of MSC-sEV flotation densities following fractionation with iodixanol density gradient. **c** Transmission electron microscopy of negative stained MSC-sEVs. Scale bar represents 100 nm. **d** Western blot analysis of MSC cell extracts (CELL), conditioned media (CM), and small exosome-like EVs (sEVs) for EV-enriched proteins (CD81, ALIX) and major contaminant protein derived from subcellular organelles such as endoplasmic reticulum (CANX, calnexin)

To understand the nature of cytokine stimulating EV release, we further extended our EV characterization. First, EVs from primed and resting MSC CM were analyzed according to recommendations from the International Society for Extracellular Vesicles (ISEV) to ascertain that they meet essential EV characteristics [36, 44]. Indeed, a more detailed NTA sizing revealed that primed MSC-EVs were mainly below 200 nm and they floated at a density of 1.13 g/mL in an iodixanol gradient (Fig. 3a,b). In transmission electron microscopy (TEM), MSC-sEVs appeared homogeneous in size (50–100 nm) and displayed cup-like morphology observed following fixation of small vesicles such as exosomes (Fig. 3c). The presence of proteins typically enriched in EVs (i.e., the ESCRT-associated protein ALIX and tetraspanin CD81), and the absence of proteins present in other organelles (i.e., the endoplasmic reticulum protein calnexin) were confirmed by Western blot and indicated the purity of the MSC-EV preparations (Fig. 3d). Importantly, nanoparticle flow cytometry confirmed that tetraspanins CD63 and CD81 were co-expressed on primed MSC-EVs, a feature associated with endosome-derived EV (Fig. S4) [45]. The MSC marker CD90 but not CD73 was identified on MSC-sEVs, as previously described [46]. Altogether, this data documents that IFN-γ and TNF-α promote MSC release of exosome-like sEVs carrying canonical MSC markers.

### Primed MSC-EVs carry unique immunomodulatory proteins and inhibit T cell proliferation

**Fig. 4:**
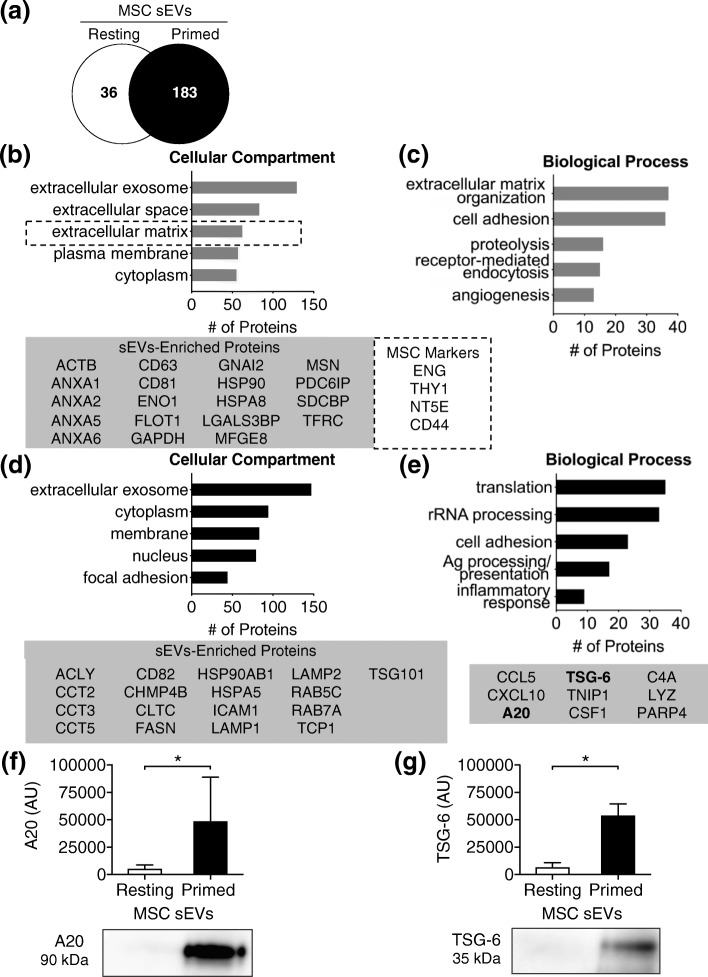
sEVs from cytokine-primed MSCs contain immunomodulatory proteins. **a** Unique and common proteins in sEVs collected from resting and primed MSC CM (*n* = 3 per group). The graph includes proteins identified in at least two samples per group. **b**, **c** Gene ontology analyses of the 161 proteins shared between resting and primed MSC-sEVs. Cellular components are shown on the left panel (**b**) and biological processes on the right panel (**c**). Proteins associated with ExoCarta’s top EV 100 proteins (http://exocarta.org), and MSC markers are listed in the boxes below (gray box lists extracellular exosome/sEV-enriched proteins and dotted line box MSC markers). **d**, **e** Cellular components (**d**, left panel) and biological processes (**e**, left panel) associated with the 183 proteins uniquely present in primed MSC-sEVs (Gene Ontology analyses). Gray box below panel (**d**) lists extracellular exosome/sEV-enriched proteins and gray box below panel (**e**) lists proteins in the “inflammatory response” category. **f, g** Western blot analysis of TSG-6 (*TNFAIP6*) and A20 (*TNFAIP3*) in resting and primed MSC-sEVs (*n* = 6). Wilcoxon signed-rank test, mean ± SD are reported, (*) represents *p* ≤ 0.05

The cargo of cytokine-primed and resting MSC-sEVs was assessed by mass spectrometry. In total, 380 proteins were identified in at least two of three samples from each group. Thirty-six of the proteins identified were only present in resting MSC-sEVs, while 183 were unique to primed MSC-sEVs. A total of 161 proteins were shared by sEVs from both groups (Fig. 4a). Analysis of cellular compartments and biological functions associated with the shared proteins and unique proteins was performed using DAVID Functional Annotation Tool. The majority of the proteins (80%, 129/161) shared between resting and primed MSC-sEVs were associated with extracellular sEVs. Among these, 19 proteins were listed in ExoCarta’s top 100 proteins that are often identified in exosomes (http://exocarta.org/sEV_markers_new). These included tetraspanins (CD63, CD81), flotillins (FLOT1), MFGE8, ALIX (PDC6IP), syntenin-1 (SDCBP), annexins (ANXA1, ANXA2, ANXA5, ANXA6), heat shock proteins (HSP90, HSPA8), and transferrin receptor (TFRC) (Fig. 4b). Proteins shared by resting and primed MSC-sEVs were involved in functions including, but not limited to, extracellular matrix organization, cell adhesion, proteolysis, receptor-mediated endocytosis, and angiogenesis.

Of the proteins uniquely identified in primed MSC-sEVs, the majority were associated with extracellular sEVs (80%, 147/183). In total, 17 of these proteins were listed in ExoCarta’s top 100 proteins found in exosomes, albeit different than those for resting MSCs. Of relevance, primed MSC-sEVs contained CD82, ESCRT-associated proteins (CHMP4B, TSG101), heat shock proteins (HSPA5, HSP90AB1), adhesion molecules (ICAM1), Rab GTPases (RAB5C, RAB7A), and lysosomal-associated membrane proteins (LAMP1, LAMP2). The unique proteins were associated with a wide range of biological processes, including, but not limited to, inflammatory response, antigen processing and presentation, cell adhesion, rRNA processing, and translation (GO biological process ontologies) (Fig. 4c). Among proteins involved in the inflammatory response, we identified candidate proteins implicated in MSC immunopotency. Among those, A20 [i.e., tumor necrosis factor-a-induced protein 3 (TNFAIP3)] and TSG-6 (i.e., TNFAIP6) (Fig. 4d) were uniquely found in sEVs from primed MSCs and not in sEVs from their resting counterparts [47, 48]. These proteins have not been previously reported in small exosome-like EVs from human AT-MSCs.

**Fig. 5:**
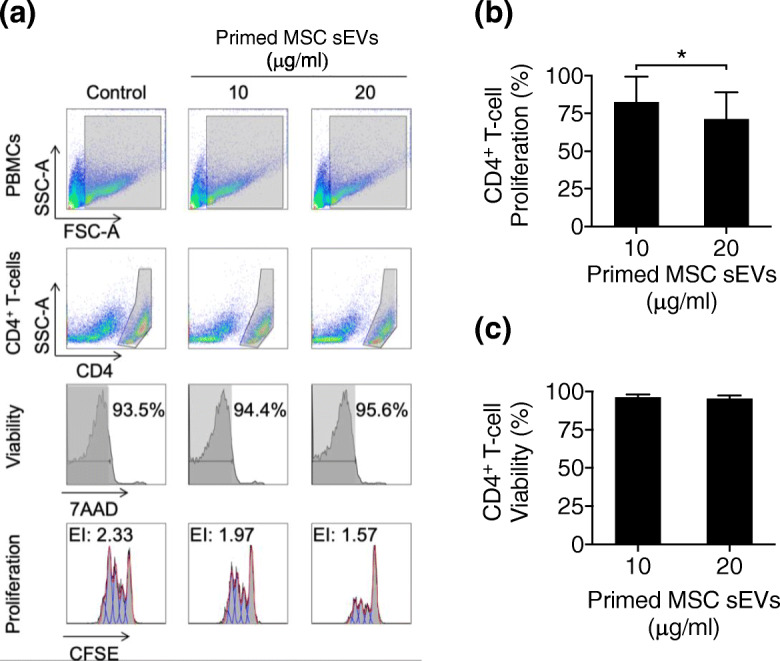
Cytokine-primed small exosome-like MSC-sEVs inhibit T cell proliferation. **a** ConA stimulated T cell proliferation assay with primed MSC-sEVs (representative example). Gating strategy and expansion index (EI) of viable CD4^+^ T cells in the presence of vehicle (Control) or increasing concentrations of primed MSC-sEVs (10 and 20 μg/ml). **b** ConA stimulated CD4^+^ T cell proliferation and **c** viability in the presence of primed MSC-sEVs. Wilcoxon signed-rank test, mean ± SD of 6 independent samples are reported, (*) represents *p* ≤ 0.05

The immunopotency of primed MSC-sEVs was assessed in an in vitro T cell proliferation assay (Fig. 5a). Primed MSC-sEVs inhibited CD4^+^ T cell proliferation in a dose-dependent manner while they did not alter T cell viability (Fig. 5b,c). In summary, cytokine-primed AT-MSCs release sEVs containing TSG-6/A20, which suppress the proliferation of activated T cells in a dose-dependent manner.

### RAB27B regulates the secretion of EVs and TSG-6 by primed MSCs

**Fig. 6:**
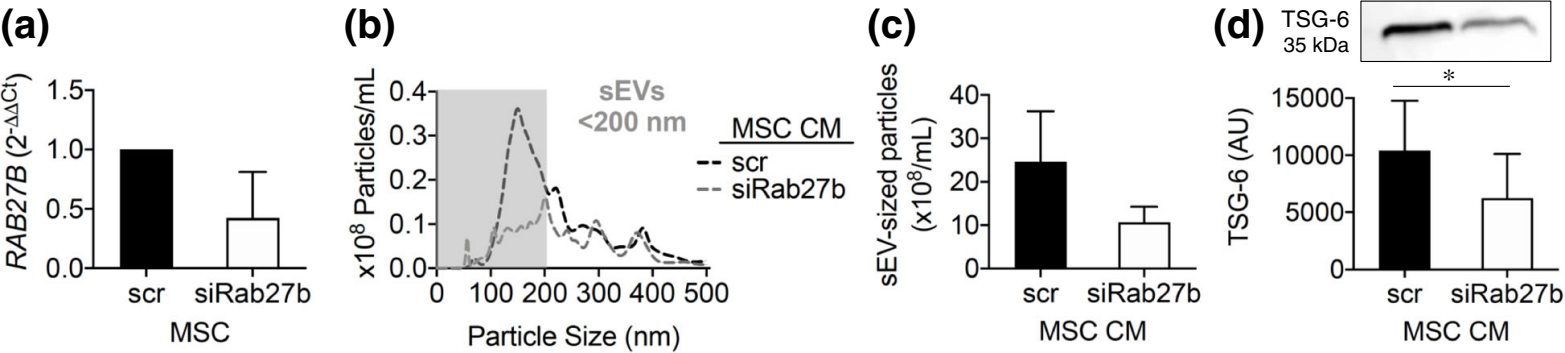
RAB27B silencing reduces the secretion of TSG-6-enriched small exosome-like EVs by primed MS. **a** *RAB27B* expression in primed MSC treated with Rab27b siRNA (siRab27b) or scrambled (scr). **b** NTA of siRab27b and control MSC CM. **c** NTA of small EVs (< 200 nm) and **d** Western blot of TSG-6 expression in CM from primed MSC treated with scr and siRab27b. In **b**, curves represent the average of 3 independent samples. The gray box indicates small EV (sEV)-sized particles (< 200 nm). In other panels, mean ± SD of three individual samples are reported; **a**, **c** non-significant differences between groups (paired *T*-test, *p* = 0.1, *p* = 0.2 respectively); in **d**, (*) represents *p* ≤ 0.05

The role of *RAB27B* on the release of sEVs by cytokine-primed MSCs was assessed in three independent experiments. *RAB27B* expression was markedly reduced in primed MSCs after treatment with *RAB27B* siRNA (Fig. 6a). This was associated with a reduction in the number of sEV-sized particles in primed MSC CM as determined by NTA (Fig. 6b,c). *RAB27B* silencing reduced TSG-6 levels in primed MSC CM (Fig. 6c). Since in the proteomic data TSG-6 was exclusively found in the cargo of primed MSC-sEVs, the results presented in Fig. 6 suggest that Rab27b may be responsible for cytokine-driven, EV-mediated release of this immunomodulatory effector.

### Primed adult MSCs secrete fewer MSC-sEVs than pediatric MSCs

**Fig. 7:**
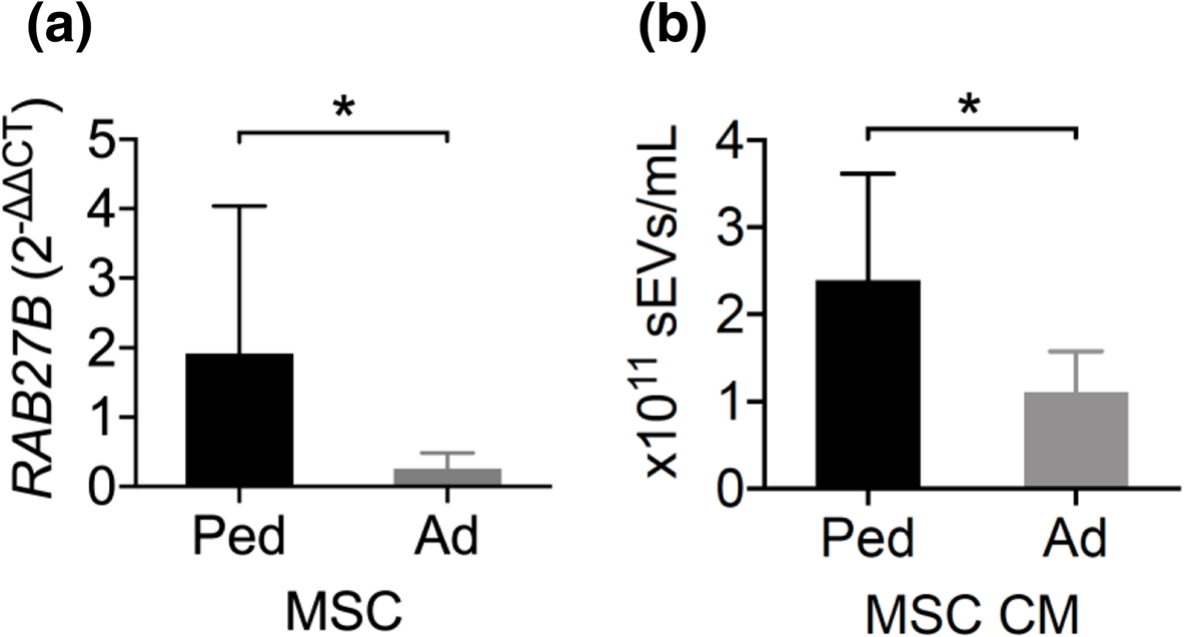
Post-priming adult MSCs secrete less small exosome-like EVs than pediatric MSCs. **a** *RAB27B* expression of primed MSC and **b** NTA of sEVs in primed MSC CM from pediatric (Ped; *n* = 9) and adult (Ad; *n* = 9) donors. Mann-Whitney test, mean ± SD are reported, where (*) represents *p* ≤ 0.05

MSCs from older donors are less efficient at suppressing allogeneic T cell proliferation (i.e., have reduced immunopotency). This, in part, relates to the fact that they secrete higher levels of “senescence-associated” pro-inflammatory cytokines [29]. We examined if MSC donors’ age also affects the secretion of cytokine-induced sEVs. The expression of *RAB27B* in primed adult MSCs was significantly lower than in pediatric MSCs (Fig. 7a). This was associated with a reduction in small exosome-like EV quantities in adult MSC CM (Fig. 7b). These results suggest that donor’s age influences the EV pathway responses to IFN-γ and TNF-α priming.

## Discussion

A well-defined property of MSCs is their capacity to secrete soluble factors and EVs (i.e., MSC secretome) that recapitulate MSC’s effects on immune responses, angiogenesis, and fibrosis [14, 29]. The composition of the secretome varies according to the MSC source, donor, and activation state among other factors. However, the contribution of the individual components of the MSC secretome to the functional effects of these cells in vivo is unknown. A clear demonstration of the mechanisms that underlie MSC’s immunomodulatory effects and those that lead to interindividual differences in biological function is key to optimizing clinical responses to MSC therapy.

Here, we studied the effect of cytokine priming on MSC-EV biogenesis, and the implication of those EVs in MSC immunopotency. Cytokine priming enhanced the immunomodulatory properties of human AT-MSC secretome and enriched it with sEVs. Using a similar mode of activation, a study on BM-MSCs found that IFN-γ and TNF-α reduced the secretion of MSC-EVs [46]. These contradictory results are likely explained by differences in the MSC tissue source and MSC-EV preparation. BM and AT-MSCs are not functionally equivalent. AT-MSCs have greater proliferative potential, secretion of cytokines, and immunomodulatory effects [49, 50]. It is not known whether vesiculation mechanisms in AT and BM-MSCs differ. The absence of serum in our study may have also influenced the quantity and cargo of MSC-EVs, as it is known that nutrient status and cellular stress can influence sEV production [51, 52]. For instance, glucose deprivation increases the quantity, repertoire of protein species, and angiogenic effects of cardiomyocyte sEVs [53]. The use of serum-free EV isolation media is key to prevent contamination with EVs present in FBS [9, 54, 55]. Moreover, FBS EVs contain RNA and can influence the activity of recipient cells [56]. Currently, there is no reliable method to completely eliminate EVs from FBS and therefore using serum-free media can be viewed as more conclusive.

The data we present support cytokine priming as a promising approach for generating bioactive AT-MSC-sEVs (small EVs) for clinical applications. Primed MSCs significantly upregulated Rab27b. This small GTPase acts as the regulator of endosome trafficking that is implicated in the release of intraluminal vesicles from MVBs as exosomes [57]. We also identified a significant upregulation of the tetraspanin CD82 in primed MSCs, which is involved in exosomal secretion of signaling proteins such as β-catenin [58]. Thus, MSC priming may lead to release of immunomodulatory exosomes.

Recently, IL-1β priming was found to enhance the immunomodulatory properties of MSCs partially through sEV-mediated transfer of miR-146a in a mouse model of sepsis [59]. LPS stimulated MSCs to release sEVs containing let-7b, which improved the effects of MSCs on wound healing [60]. Further studies are needed to determine whether IFN-γ and TNF-α priming of AT-MSCs affects the RNA cargo of their sEVs in a manner relevant to their potential use as therapy for inflammatory conditions.

MSC-sEVs are proposed to act by delivering their cargo of proteins to the recipient cells [61]. Moreover, the MSC environment (e.g., hypoxic conditions) and specific interventions (e.g., radiation, nutrient starvation) impact the proteomic profile of MSC-EVs and, as a consequence, their functional effects [62–65]. Herein, we provide a detailed proteomic analysis of cytokine-induced sEVs enriched in small exosome-like EVs from human AT-MSCs. We demonstrate that the proteomic cargo of AT-MSC-sEVs shifts in response to pro-inflammatory cytokines. TSG-6 and A20, key molecules implicated in the immunopotency of MSCs, are both part of the molecular content of cytokine-primed AT-MSC-sEVs.

TSG-6 is a multi-functional protein involved in several anti-inflammatory processes, including the inhibition of neutrophil transendothelial migration and chemotaxis; the shift of macrophages to an anti-inflammatory M2 phenotype; and the inhibition of T cell activation and IFN-γ secretion [48, 66, 67]. TSG-6 secretion has been shown to be necessary for MSC’s immunomodulatory effects in several models of inflammation (e.g., cornea injury, peritonitis, myocardial infarction, and lung injury). Umbilical cord-derived MSC (UC-MSC) EVs contain TSG-6, which is responsible for their therapeutic effects in experimental bronchopulmonary dysplasia [68]. This emphasizes the importance of TSG-6 in mediating the anti-inflammatory effects of MSC-EVs. Our study shows that primed (but not resting) AT-MSCs may use EV biogenesis pathway to release TSG-6 to the microenvironment.

A20 is a cytoplasmic protein that terminates nuclear factor-κB (NF-κB) signaling [69]. Mutations in A20/TNFAIP3 locus have been associated with inflammatory diseases/conditions such as rheumatoid arthritis, systemic lupus erythematosus, and coronary artery disease [70–72]. In the absence of A20, transgenic mice display LPS/TNF hypersensitivity and are unable to limit inflammation, resulting in premature death [73]. The importance of A20 to the immunosuppressive properties of MSCs has only been demonstrated once in a mouse melanoma model [47]. More evidence is needed to understand the mechanism by which MSC-derived A20 acts on recipient immune cells. To date, A20 has only been identified in EVs secreted by TNF-α-treated endothelial cells; the functional importance of which is not yet known [74]. Herein, we provide evidence that AT-MSCs secrete A20 into the extracellular space via small exosome-like EVs in response to IFN-γ and TNF-α. Given the importance of TSG-6 and A20 in immunomodulation, these proteins could serve as useful biomarkers for the selection of anti-inflammatory MSC-sEVs for clinical applications.

Many published studies that previously evaluated the in vitro anti-inflammatory effects of MSC-EVs yielded inconsistent results due to differences in MSC sources, modes of EV collection, and in vitro potency assay designs [75–78]. We show that cytokine-primed AT-MSCs produce sEVs that can inhibit the proliferation of ConA-stimulated CD4^+^ T cells at concentrations of 10 and 20 μg/ml. Taking into account that the sEV protein yield per million MSCs was 15 ± 7.4 μg (*n* = 7), the suppressive capacity of MSC-sEVs is significantly less than that exerted by the parental cells or MSC CM. However, this does not exclude the possibility that sEVs work synergistically with other soluble factors to achieve greater T cell inhibition.

Rab27b is responsible for cytokine-primed MSC-EV release, and its inhibition with siRNA reduces the release of these sEVs and inhibits the secretion of the anti-inflammatory molecule TSG-6. The siRab27b treatment did not completely abrogate *RAB27B* expression in AT-MSCs; therefore, we can only speculate that a complete knockdown of Rab27b may result in full inhibition of the release of TSG-6 containing sEVs. Given that Rab27b mediated TSG-6 secretion may be essential to MSC immunopotency, future studies should test whether *RAB27B* measurement may predict MSC-EV production and their capacity to reduce inflammation [48]. Recent reports implicate Rab27-dependent sEV production in regulating immunity [79]. In the absence of RAB27A/B, mice develop chronic low-grade inflammation and are not able to mount an immune response to inflammatory stimuli, i.e., lipopolysaccharide (LPS) [79]. Further studies are needed to confirm whether MSC-EV release and TSG-6/A20 secretion is abrogated in RAB27 double knock-out mice and whether defects in this inflammation-resolving mechanism contribute to accelerated aging (“inflamm-aging”) [80, 81].

In this paper, we show differences in *RAB27B* expression and small exosome-like EV secretion between MSC pediatric and adult donors. Age was associated with lower RAB27B expression and sEV secretion by MSCs. This points to a new role of RAB27B and is consistent with previous data indicating that developmental maturity (embryonic stem cell [ESC]- vs UC-MSC) is inversely correlated with sEV yield [82]. We report that RAB27B mediates the release of anti-inflammatory sEVs by primed MSCs and that lower RAB27B expression is associated with age. *RAB27B* screening may therefore be tested as a predictor of clinical outcomes post-MSC treatment of inflammatory conditions.

## Conclusions

Subcellular MSC products are evaluated as therapeutics on the basis of their potential superior safety profile and functional stability upon storage compared to MSCs. The results we present indicate that the quantity of MSC-derived EVs and their cargo vary according to the MSC activation state and the MSC donor’s age. Ultimately, these results support the notion that “not all MSC-EVs are created equal.” This concept is clinically relevant and stresses the importance of optimizing EV preparations, including cytokine priming as a way to enhance MSC-EV yields and therapeutic efficacy. In addition, our results highlight the existence of age-associated differences in MSC vesiculation and suggest that reduced immunoregulatory effects of MSCs from older donors may be linked to age-related changes in cellular EV profiles.

## Supplementary Information

**Additional file 1.**

## Data Availability

The datasets used and/or analyzed during the current study are available from the corresponding author on reasonable request.

## Abbreviations

A20: Tumor necrosis factor alpha-induced protein 3 (TNFAIP3)
ALIX: ALG-2-interacting protein X
ANX: Annexin
AT: Adipose tissue
AT-MSC: Adipose tissue derived multipotent mesenchymal stromal cells
bFGF: Basic fibroblast growth factor
BM: Bone marrow
BM-MSC: Bone marrow-derived multipotent mesenchymal stromal cells
CHMP4B: Charged multivesicular body protein 4B
CM: Conditioned media
ConA: Concanavalin A
EGF: Epidermal growth factor
EVs: Extracellular vesicles
ESCRT: Endosomal sorting complex required for export
FBS: Fetal bovine serum
FLOT1: Flotilin 1
HGF: Hepatocyte growth factor
HSP: Heat shock protein
ICAM1: Intercellular adhesion molecule 1 (CD54)
IFN-γ: Interferon gamma
IGF-1: Insulin-like growth factor 1
IL: Interleukin
LAMP: Lysosomal-associated membrane proteins
MFGE8: Milk fat globule—epidermal growth factor—factor VIII (lactadherin)
MSC: Multipotent mesenchymal stromal cells
MSC CM: Multipotent mesenchymal stromal cell-conditioned media
MSC-EVs: Multipotent mesenchymal stromal cell-derived extracellular vesicles
NF-κB: Nuclear factor kappa B
PDC6IP: Programmed cell death 6-interacting protein
PGE_2_: Prostaglandin E2
RAB27B: Member RAS oncogene family
RI-MUHC: Research Institute of the McGill University Health Centre
SDCBP: Syndecan binding protein
sEVs: Small extracellular vesicles
siRNA: Small interfering RNA
TEM: Transmission electron microscopy
TFRC: Transferrin receptor
TGF-β1: Transforming growth factor beta 1
TNF-α: Tumor necrosis factor alpha
TSG-6: Tumor necrosis factor alpha-inducible Protein 6 (TNFAIP6)
VEGF: Vascular endothelial growth factor
