# Supplementary material for: Human multipotent mesenchymal stromal cells cytokine priming promotes RAB27B-regulated secretion of small extracellular vesicles with immunomodulatory cargo
Source: Stem Cell Res Ther. 2020 Dec 14;11:539. doi: 10.1186/s13287-020-02050-6 (PMC7734842; doi:10.1186/s13287-020-02050-6)
Supplement: Supplementary file 1 — Additional file 1. [file 13287_2020_2050_MOESM1_ESM.docx]

## Supplemental Figures


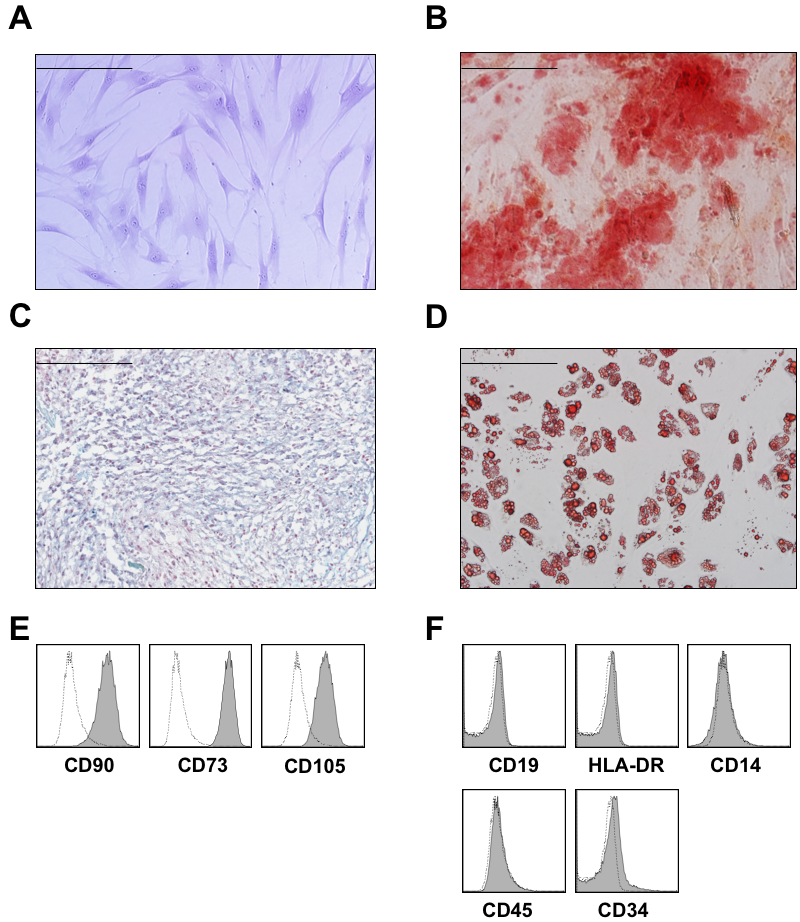


### Figure S1: Minimal criteria for defining multipotent mesenchymal stromal cells (MSCs)

**(A)** Crystal violet stained MSCs displaying plastic-adherence and spindle-shaped morphology. **(B)** Oil red O-stained adipocytes. **(C)** Alcian blue-stained chondroblasts. **(D)** Alizarin red-stained osteoblasts. **(E-F)** Flow cytometry analysis of MSCs surface marker expression (CD73, CD90, and CD105) and lack of CD19, HLA-DR, CD14, CD45, and CD34. Bars represent 200 µm.

**A B**


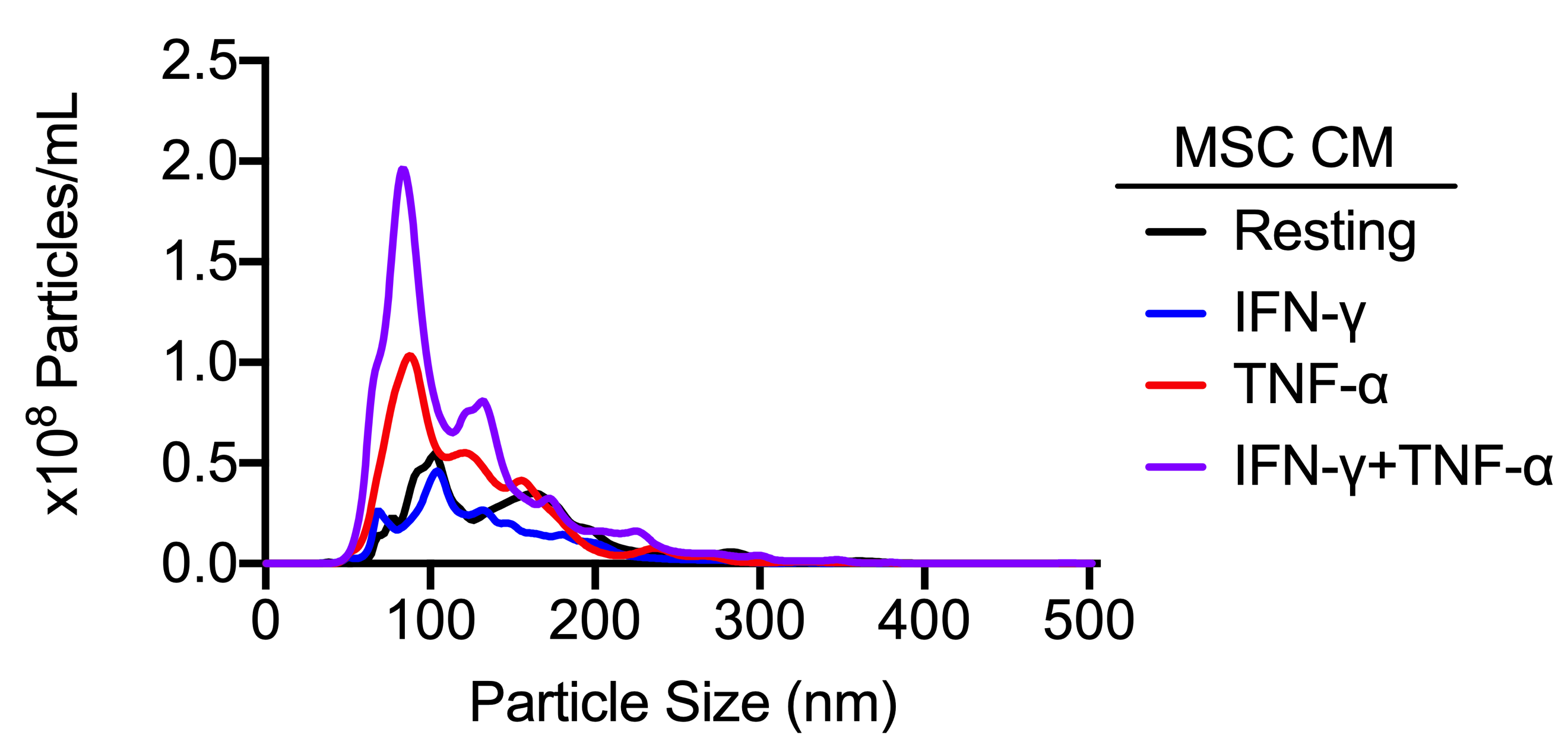

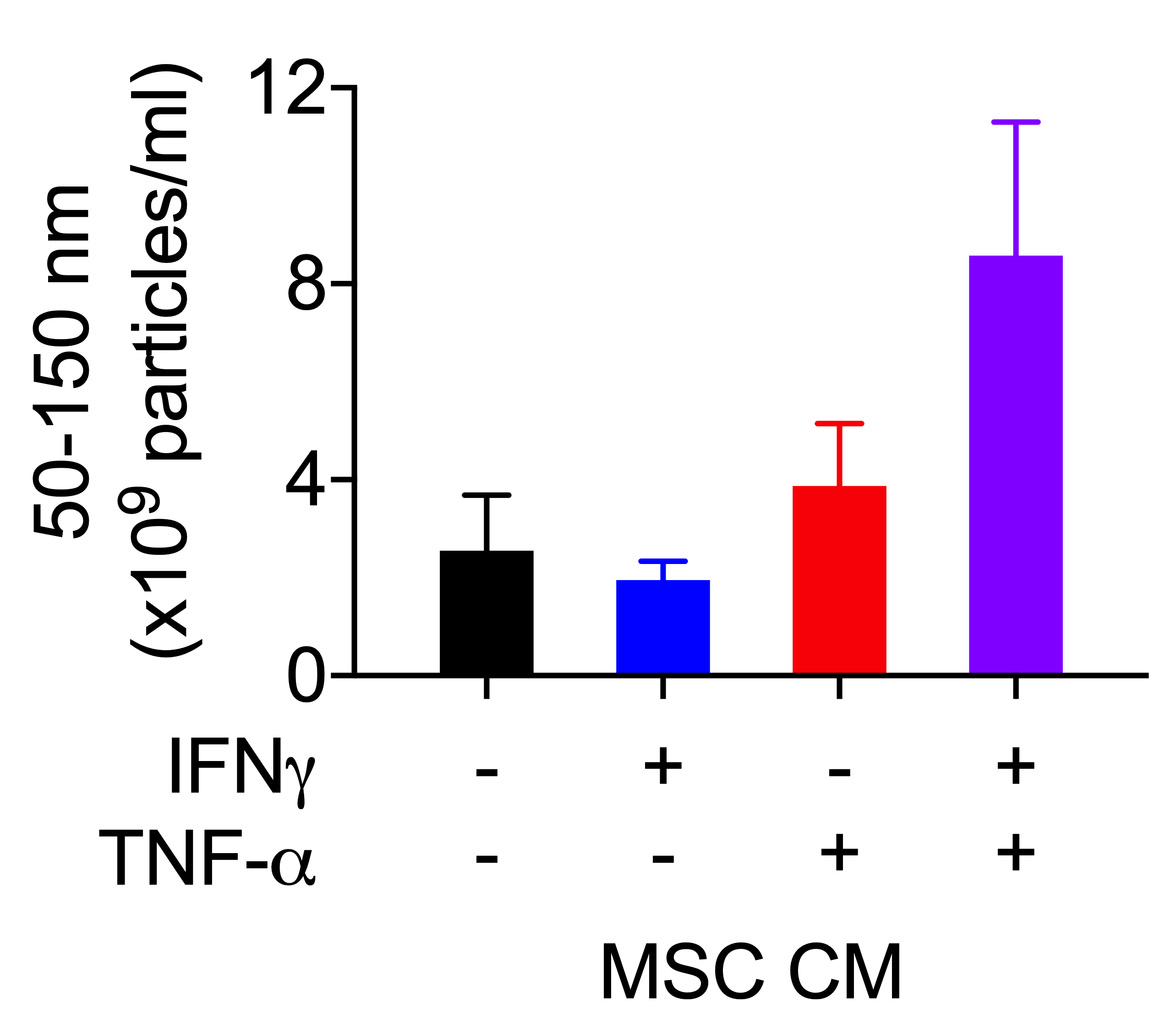


*

### Figure S2: Priming-induced MSC sEVs release was specific to IFN-γ and TNF-α treatment and not to each of these cytokines independently

(A) Distribution of nanoparticles and (B) Summary small EV-sized particles (50-150 nm) in MSC CM after activation with 10 ng/ml IFN-γ and/or 15 ng/ml TNF-α for 72 hours. Mean±SD of four individual samples are reported; (*) represents p≤0.05.

**A B**

**
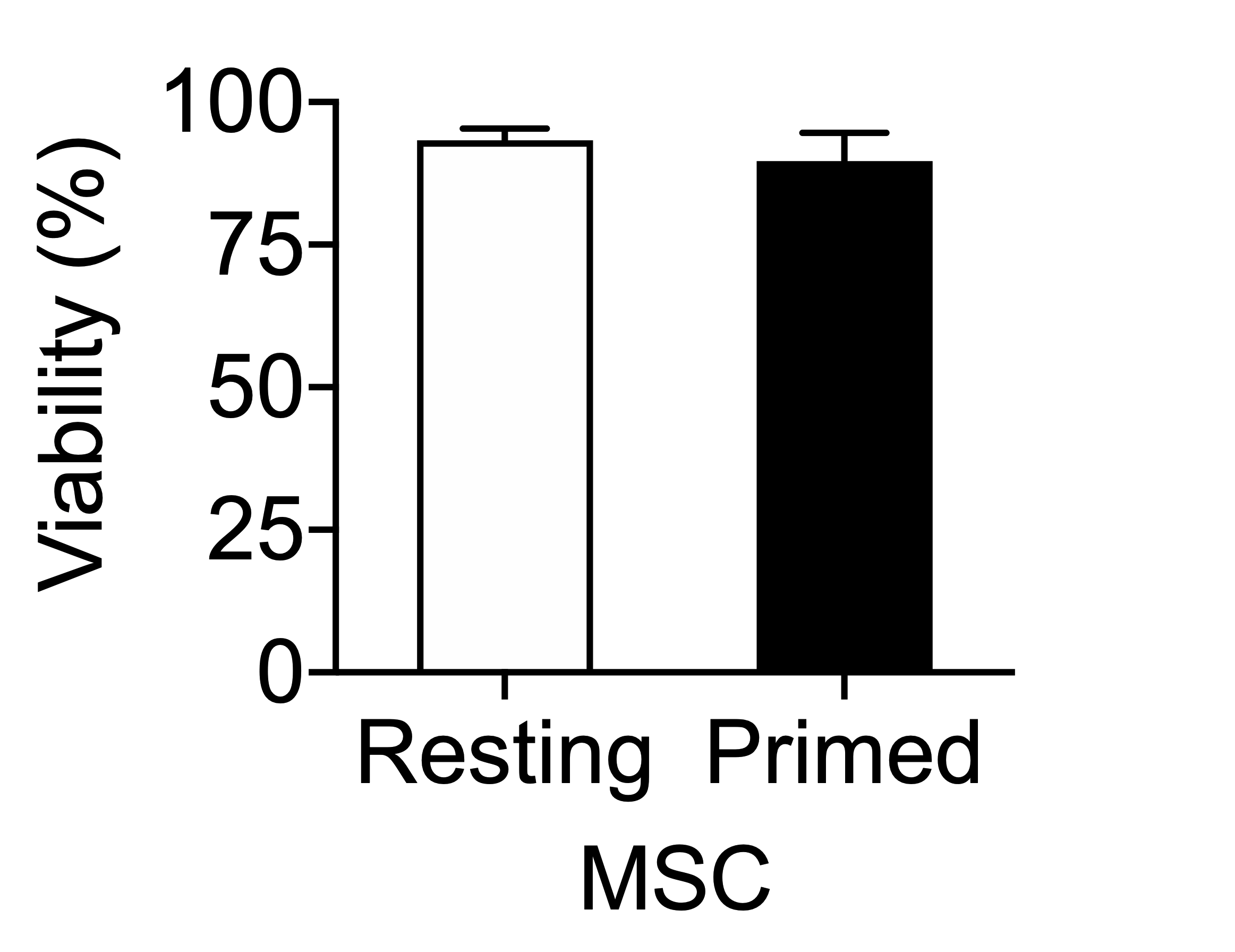
**  MSC Singlets Viability

**
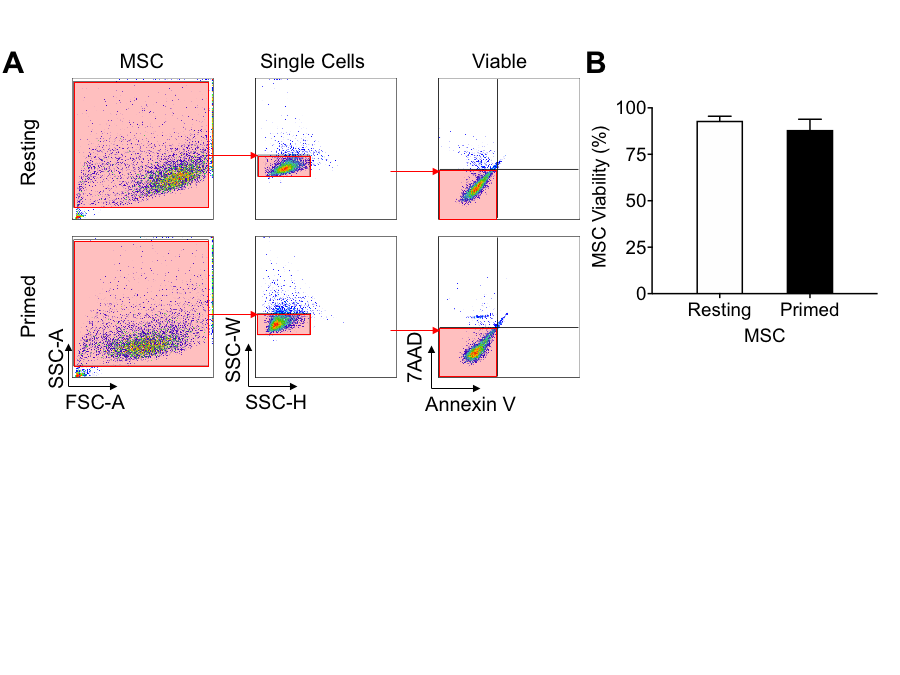
**

### Figure S3. Cytokine-priming does not affect MSC viability

Viability (Annexin V^-^/7AAD^-^) of resting and cytokine-activated (10 ng/ml IFN-γ and 15 ng/ml TNF-α for 72 hours) MSCs (n=3).

**A B**


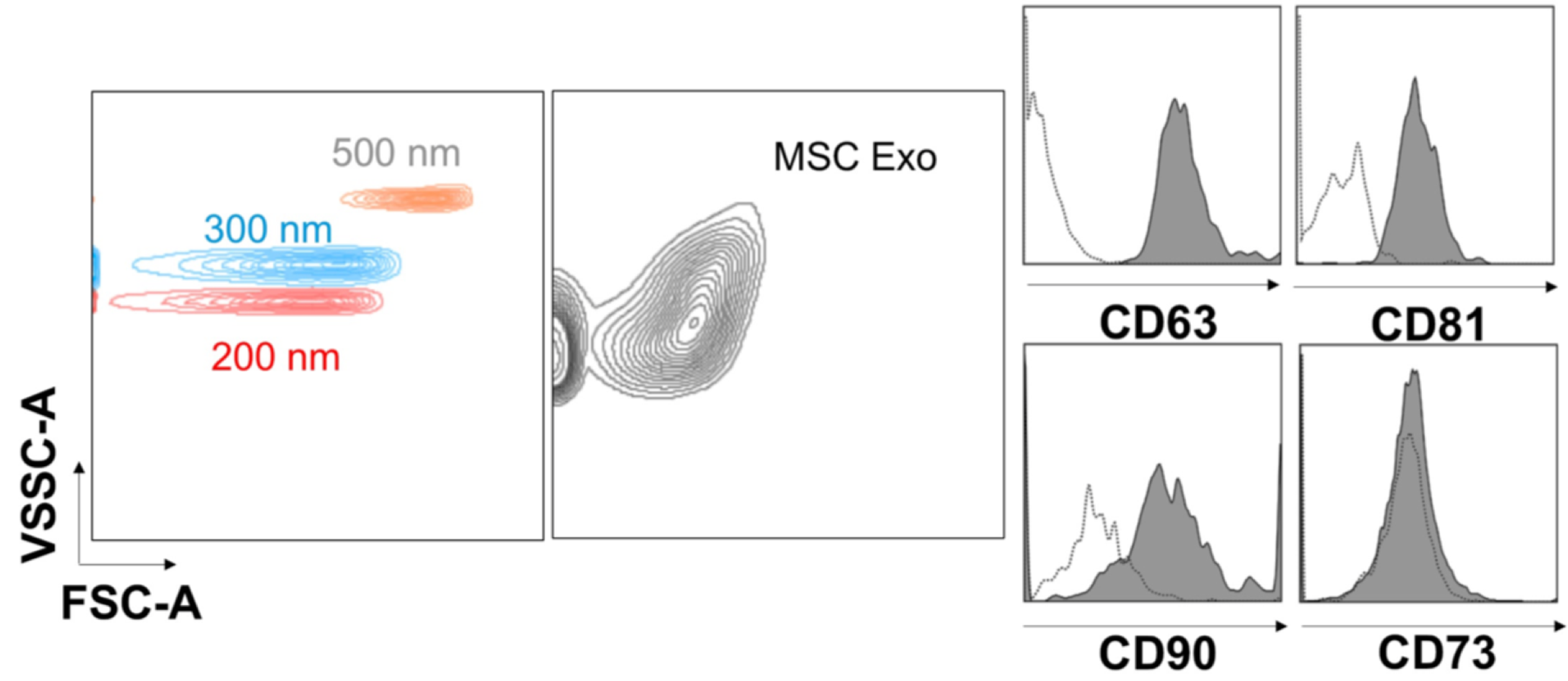


MSC sEVs

### Figure S4: MSC sEVs co-express CD63, CD81 and CD90.

Flow cytometry evaluation of MSC sEVs size distribution and co-expression of EVs (i.e. CD63, CD81) and MSC markers (i.e. CD90, CD73).

## Supplemental Tables

| Table S1. Demographics of MSC donors | | |
| --- | --- | --- |
|  | Pediatric | Adult |
| Number of subjects | 9 | 14 |
| Sex, (female/male) | 5/4 | 8/6 |
| Age, mean ± SD years | 16.1±2.6 | 62.6±12.7 |
| Ethnicity, n (%) |  |  |
| African American | 1 (11.1) | 1 (7.1) |
| Caucasian | 8 (88.9) | 10 (71.4) |
| Asian | 0 (0.0) | 3 (21.4) |

## Supplemental Experimental Procedures

### MSC Multi-lineage Differentiation

To assess the osteogenic and adipogenic potential, P4 MSCs were seeded in 24-well plates at a density of 5,000 cells/cm^2^. After four days, the media was replaced with differentiation medium (Gibco™ StemPro® adipogenesis or osteogenesis differentiation kit, Thermo Fisher Scientific) or complete medium, and replenished every 3-4 days for 20 days. MSCs were fixed with 4% formaldehyde and stained with Alizarin Red S or Oil Red O (Electron Microscopy Sciences) to evaluate osteogenesis and adipogenesis respectively. For chondrogenic differentiation, a micromass pellet of 250,000 MSCs was expanded in a 24-well plate for 20 days with Gibco™ StemPro® chondrogenesis differentiation medium (Thermo Fisher Scientific, Waltham, MA), fixed, sectioned (1 μm), and stained with Alcian Blue.

### MSC Surface Markers

The expression of surface markers on MSCs was determined by multiparametric flow cytometry (BD LSRII; Becton Dickinson Co). P4 MSCs were stained with the following fluorochrome-conjugated monoclonal antibodies (BD Biosciences): fluorescein isothiocyanate (FITC)-conjugated anti-CD90 (555595) and anti-CD45 (555482); phycoerythrin (PE)-conjugated anti-CD73 (555749); allophycocyanin (APC)-conjugated anti-CD34 (555824), anti-CD19 (555415) and anti-HLA-DR (560896); peridinin chlorophyll (PerCP)-conjugated anti-CD105 (560819), and anti-CD14 (562692). Data analysis was performed using FlowJo software 9.7.2.

### Nanoparticle Tracking Analysis of IFN-γ and/or TNF-α-primed MSC CM

2.5x10^5^ MSC were seeded in 24 well plates in 1 ml of complete medium (1.0g/L glucose, with L-glutamine & sodium pyruvate Dulbecco’s modified Eagle’s medium [DMEM, WISENT Inc.) supplemented with 10% FBS and 1% penicillin/streptomycin (10,000 units/mL penicillin, 10,000 mg/mL streptomycin, WISENT Inc.). After 24 hours, the wells were washed with PBS and replaced with 1ml of sEVs isolation media (phenol red-free low glucose DMEM containing 1% insulin-transferrin-selenium (ITS, Thermo Fisher Scientific)) with 10 ng/ml IFN-γ and/or 15 ng/ml TNF-α. Resting MSCs (no cytokines added) were used as controls. After 72 hours, MSC CM was collected and the size and concentration of nanoparticles was measured using NanoSight NS500 (NanoSight). PBS was used to dilute MSC CM (1:12) to achieve 40-100 particles per frame during quantification. Five 30 second videos were obtained at room temperature for each sample.

### MSC Viability

Resting and primed MSCs were stained with Annexin V (556421) and 7-Aminoactinomycin D (7-AAD) (559925) in binding buffer (BD Biosciences). Data analysis was performed using FlowJo software 9.7.2.

### MSC sEVs Surface Markers

The expression of surface markers on MSC-sEVs was determined by multiparametric flow cytometry (CytoFLEX, Beckman Coulter) as previously described (1). Briefly, MSC-sEVs were suspended in 0.02 µm-filtered PBS and stained with a combination of the following fluorochrome-conjugated monoclonal antibodies (BD Biosciences): FITC-conjugated anti-CD90 (555595); PE-conjugated anti-CD73 (555749) or FITC-conjugated anti-CD63 (557305); and APC-conjugated anti-CD81 (561958). The samples were incubated in the dark for 15 minutes before centrifugation to remove excess antibodies (2,000g for 10 min). Non-labeled sEVs and antibodies suspended in filtered PBS were used to discriminate positive and negative populations. A total of 100,000 events were acquired. Calibration beads (Megamix-Plus FSC) suspended in PBS were used to discriminate 200, 300 and 500 nm particles using the FSC parameter. Data was analyzed with FlowJo software 10.4.1.

## Supplemental References

1. Ramos TL, Sánchez-Abarca LI, Muntión S, Preciado S, Puig N, López-Ruano G, et al. MSC surface markers (CD44, CD73, and CD90) can identify human MSC-derived extracellular vesicles by conventional flow cytometry. Cell Communication and Signaling. 2016;14(1):2.
